# Crystal structure and Hirshfeld surface analysis of 4′-(2-chloro­phen­yl)-1′-methyl-3′′-phenyl-7′′,8′′-di­hydro-5′′*H*-di­spiro­[indoline-3,2′-pyrrolidine-3′,6′′-iso­quinoline]-2,5′′-dione

**DOI:** 10.1107/S2056989018005455

**Published:** 2018-04-12

**Authors:** R. Vishnupriya, C. Selva Meenatchi, J. Suresh, R. V. Sumesh, R. Ranjith Kumar, P. L. Nilantha Lakshman

**Affiliations:** a Department of Physics, The Madura College, Madurai 625 011, India; b Department of Organic Chemistry, School of Chemistry, Madurai Kamaraj University, Madurai 625 021, India; c Department of Food Science and Technology, University of Ruhuna, Mapalana, Kamburupitiya 81100, Sri Lanka

**Keywords:** crystal structure, di­spiro, indoline, pyrrolidine, iso­quinoline, hydrogen bonding, Hirshfeld surface analysis

## Abstract

In the title di­spiro compound, C_32_H_26_ClN_3_O_2_, the cyclo­hexa­none ring of the iso­quinoline unit has a distorted envelope conformation, with the methyl­ene C atom adjacent to the spiro C atom as the flap. The central 1-methyl­pyrrolidine ring has an envelope conformation with the N atom as the flap. The mean planes of the indolin-2-one ring system, the chloro­benzene ring and the iso­quinoline ring system are inclined to the mean plane of the central 1-methyl­pyrrolidine ring by 87.95 (11), 71.01 (12) and 88.81 (10)°, respectively. There are two short C—H⋯O intra­molecular contacts present. In the crystal, mol­ecules are linked *via* C—H⋯ O hydrogen bonds, forming chains along the *a*-axis direction. The NH H atom is involved in a weak N—H⋯O hydrogen bond with the same carbonyl O atom. There are no further significant inter­molecular contacts present. The largest contribution to the overall Hirshfeld surface of 52.3% is due to H—H contacts.

## Abstract 2

In the crystal structure of the title compound, a di­spiro­[indoline-3,2′-pyrrolidine-3′,6′′-iso­quinoline]-2,5′′-dione, C—H⋯O hydrogen bonding predominates, linking mol­ecules to form chains propagating along [100].

## Chemical context   

Spiro scaffolds are being used more and more in drug discovery because of their built-in three-dimensionality and structural variations, resulting in new synthetic routes to introduce spiro building blocks into more pharmaceutically active mol­ecules (Kobayashi *et al.*, 1991 ▸; James *et al.*, 1991 ▸). The spiro-pyrrolidine ring system is a structural motif present in many biologically important and pharmacologically relevant alkaloids. Spiro-pyrrolidine-indolin-2-one ring systems are also found in a number of alkaloids of biological importance (Hilton *et al.*, 2000 ▸). Some derivatives are used as anti­microbial and anti­tumour agents (Sundar *et al.*, 2011 ▸), or possess analgesic (Crooks & Sommerville, 1982 ▸) and anti-influenza virus (Stylianakis *et al.*, 2003 ▸) activities. In view of this importance, the primary goal for the X-ray analyses of the title compound is to obtain detailed information on the structural conformation that may be useful in understanding the chemical reactivity of such compounds.

## Structural commentary   

**Figure 1:**
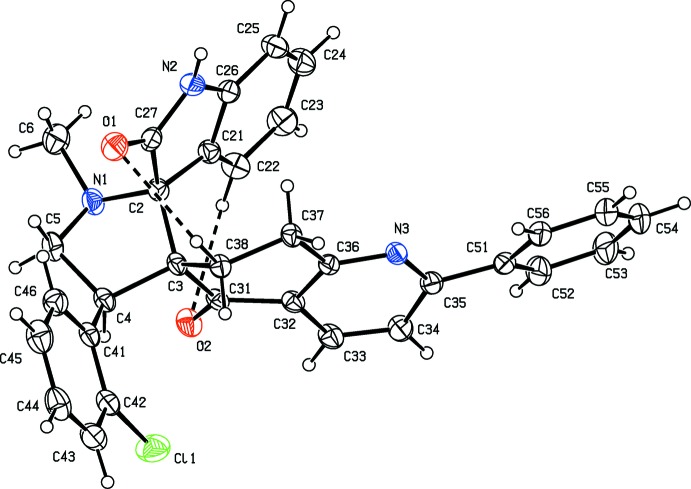
The mol­ecular structure of the title compound, showing 30% probability displacement ellipsoids and atom labelling. The intra­molecular C—H⋯O contacts (see Table 1 ▸) are shown as dashed lines.

The mol­ecular structure of the title mol­ecule is shown in Fig. 1 ▸. There are two short C—H⋯O intra­molecular contacts present (Table 1 ▸). In the iso­quinoline ring system (N3/C3/C31–C38) the cyclo­hexa­none ring (C3/C31–C38) adopts a distorted envelope conformation [puckering parameters: *Q* = 0.500 (2) Å, θ = 63.7 (2)°, φ = 308.9 (3)°], with atom C38 as the flap. The pyridine ring (N3/C32–C36) has a shallow twist-boat conformation [puckering parameters: *Q* = 0.094 (2) Å, θ = 92.3 (13)°, φ = 84.5 (13)°]. Their mean planes are inclined to each other by 14.06 (10)°, and the phenyl ring (C51–C56) is inclined to the pyridine ring mean plane by 22.35 (12)°.
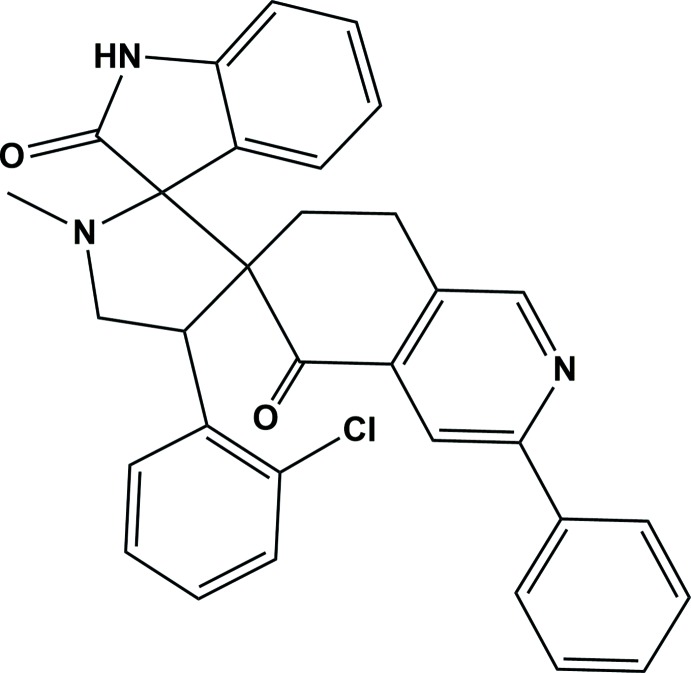


In the indolin-2-one ring system (N2/C2/C21–C27), the benzene (C21–C26) and pyrrolidine (N2/C2/C21/C26/C27) rings make a dihedral angle of 2.45 (12)°, while the keto atom O1 deviates from the attached pyrrolidine ring by 0.043 (1) Å. The 1-methyl­pyrrole ring (N1/C2–C5) has an envelope conformation with atom N1 as the flap [puckering parameters: *Q* = 0.094 (2) Å, θ = 92.3 (13)°, φ = 84.5 (13)°]. The mean planes of the indolin-2-one ring system, the chloro­benzene (C41–C46) ring and the iso­quinoline (N3/C3/C31–C38) ring system are inclined to the mean plane of the central 1-methyl­pyrrolidine (N1/C2–C5) ring by 87.95 (11), 71.01 (12) and 88.81 (10)°, respectively. The sum of the bond angles around atoms N1 and N2 are 333.6 and 358.6°, respectively, indicating a pyramidal geometry and sp^3^ hybridization.

## Supra­molecular features   

**Figure 2:**
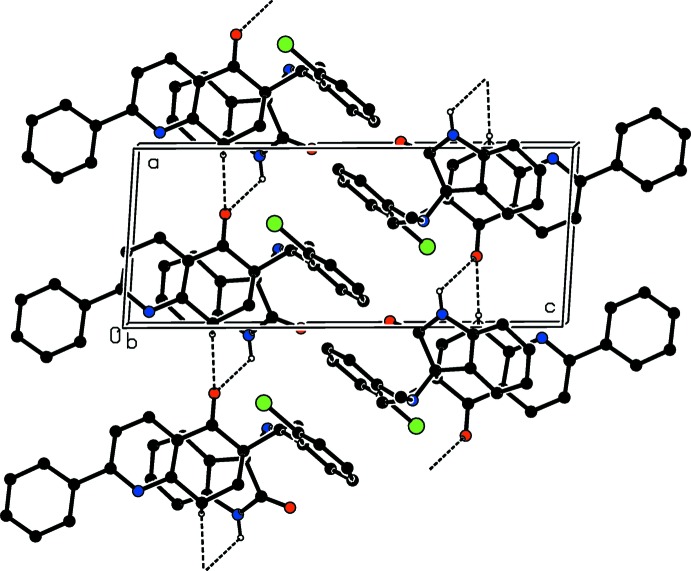
A view along the *b* axis of the crystal packing of the title compound, illustrating the formation of the hydrogen-bonded (dashed lines; Table 1 ▸) chains running along the *a*-axis direction. H atoms not involved in these inter­actions have been omitted for clarity.

In the crystal, mol­ecules are linked by C—H⋯O hydrogen bonds and a weak N—H⋯O hydrogen bond, forming chains propagating along the *a-*axis direction (Fig. 2 ▸ and Table 1 ▸). There are no further significant inter­molecular inter­actions present.

## Database survey   

**Figure 3:**
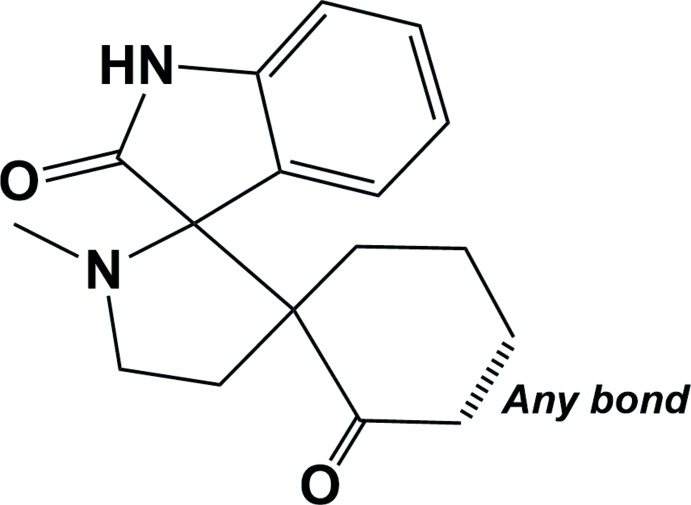
Structural fragment for the CSD search.

A search of the Cambridge Structural Database (Version 5.39, last update February 2018; Groom *et al.*, 2016 ▸) for the central di­spiro fragment, 1′-methyl­dispiro­[cyclo­hexane-1,3′-pyrrolidine-2′,3′′-indoline]-2,2′′-dione (see Fig. 3 ▸), gave eight hits of which coordinates were available for six structures. Two compounds closely resemble the title compound, *viz.* 4′-(4-chloro­phen­yl)-1′-methyl-3,4-di­hydro-1*H*-di­spiro­[acridine-2,3′- pyrrolidine-2′,3′′-indole]-1,2′′(1′′*H*)-dione methanol solvate (CSD refcode NAQCAL: Maheswari *et al.*, 2012 ▸), and 4′-(2,4-di­chloro­phen­yl)-1′,3′′-dimethyl-1′′-phenyl-7′′,8′′-di­hydro­dispiro­[indole-3,2′-pyrrolidine-3′,6′′-pyrazolo­[3,4-*b*]quinoline]-2,5′′(1*H*,1′′*H*)-dione chloro­form solvate (UQIROD; Sumesh *et al.*, 2016 ▸). In both compounds, the mean plane of the 1-methyl­pyrrolidine ring was found to be almost perpendicular to the mean plane of the indoline ring system and the mean plane of the cyclo­hexa­none ring, similar to the situation in the title compound, see Section 2 *Structural commentary.*

## Hirshfeld Analysis   

The program *CrystalExplorer* (Wolff *et al.*, 2012 ▸) was used to generate the Hirshfeld surfaces mapped over *d*_norm_, and the electrostatic potential for the title compound. The contact distances, *d*_i_ and *d*_e_, from the Hirshfeld surface to the nearest atom, inside and outside, respectively, enable the analysis of the inter­molecular inter­actions through the mapping of *d*_norm_. Two-dimensional fingerprint plots (Rohl *et al.*, 2008 ▸) provide an indication of the inter­molecular contacts in the crystal.

**Figure 4:**
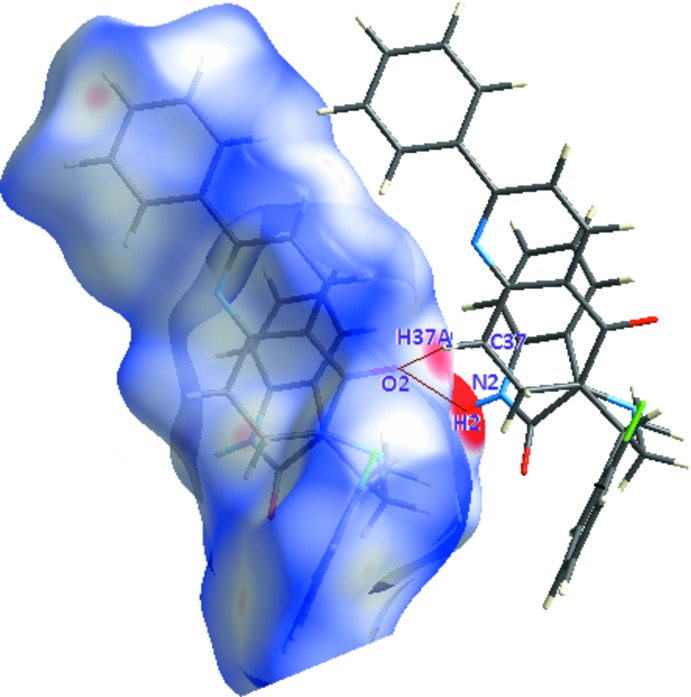
*d*_norm_ mapped on the Hirshfeld surface for visualizing the contacts of the title compound. Dotted lines indicate hydrogen bonds.

The hydrogen-bonding network generated in the crystal can be visualized using Hirshfeld surface analysis. The bright-red spots on the Hirshfeld surface mapped over *d*_norm_ (Fig. 4 ▸), with labels H2 and H37*A*, on the surface represent donors for potential hydrogen bonds (see Table 1 ▸); the corresponding acceptor on the surface appears as a bright-red spot at atom O2.

**Figure 5:**
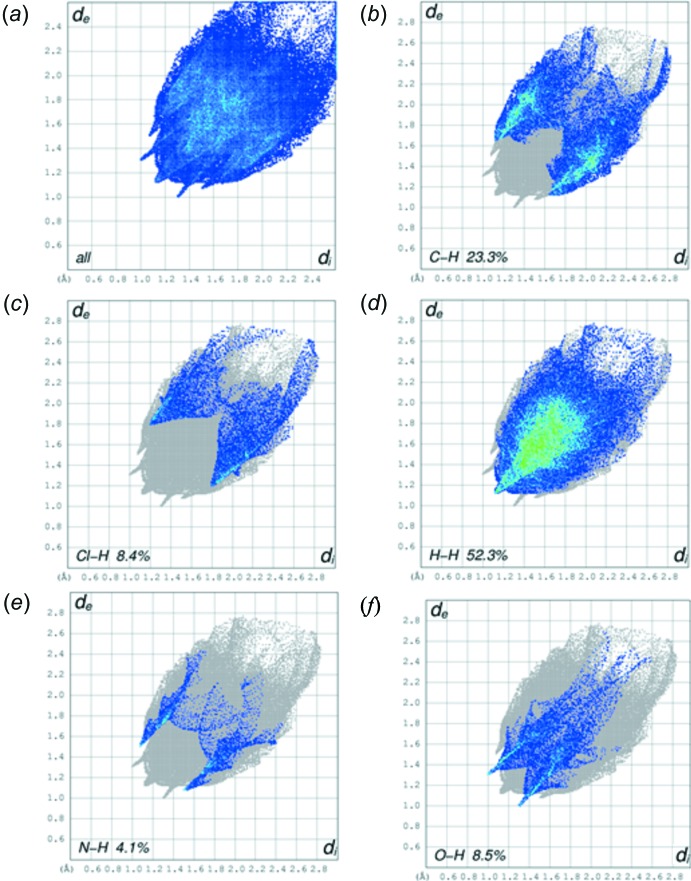
Fingerprint plot of the title compound, (*a*) all, (*b*) H⋯H, (*c*) C⋯H/H⋯C, (*d*) O⋯H/H⋯O, (*e*) Cl⋯H/H⋯Cl and (*f*) N⋯H/H⋯N contacts. The outline of the full fingerprint plots is shown in grey. *d*_i_ is the closet inter­nal distance from a given point on the Hirshfeld surface and *d*_e_ is the closest external contact.

The overall two-dimensional fingerprint plot is illustrated in Fig. 5 ▸ *a*, and those delineated into C⋯H/H⋯C, Cl⋯H/H⋯Cl, H⋯H, N⋯H/H⋯·N and O⋯H/H⋯O in Fig. 5 ▸ *b*–*f*, respectively. The greatest contribution to the overall Hirshfeld surface, *i.e*. 52.3%, is due to H⋯H contacts (Fig. 5 ▸ *d*; widely scattered points with a high concentration in the middle region, shown in green). The relative contributions of the other different inter­molecular inter­actions to the Hirshfeld surface in descending order are: C⋯H/H⋯C (23.3%), O⋯H/H⋯O (8.5%), Cl⋯H/H⋯Cl (8.4%), N⋯H/H⋯N (4.1%) and there is only a very small contribution from other contacts, *i.e*. 3.1%, in the structure. This illustrates that the N—H⋯O and C—H⋯O inter­actions contribute significantly to the crystal packing of the title compound.

## Synthesis and crystallization   

An equimolar mixture of 2-phenyl-5,6,7,8-tetra­hydro-5-quinolinone and 2-chloro­benzaldehyde was dissolved in 10 ml of ethanol followed by the addition of 0.5 equiv. of potassium hydroxide. The mixture was stirred for 1 h at ambient temperature and the precipitate formed was filtered and dried to obtain pure (*E*)-6-(2-chloro­benzyl­idene)-2-phenyl-7,8-di­hydro­quinolin-5(6*H*)-one (*L*) in 94% yield (m.p. 323–324 K). A mixture of isatin (1.1 mmol) and sarcosine (1.1 mmol) was taken in 10 ml of aceto­nitrile in a 50 ml round-bottom flask and heated to reflux for 2 h. Then 1 mmol of *L* was added to the above reaction mixture and reflux was continued for a further 14 h. After completion of the reaction, as evident from TLC, the solvent was removed under reduced pressure and the residue washed with ice-cold water (50 ml). The crude product was purified by column chromatography using a 90:10 (*v*/*v*) petroleum ether–ethyl acetate mixture to obtain the pure product (yield 82%, m.p. 356 K). Colourless block-like crystals were obtained by slow evaporation of a solution in ethyl acetate.

## Refinement   

Crystal data, data collection and structure refinement details are summarized in Table 2 ▸. The NH H atom was located in a difference-Fourier map and freely refined. The C-bound H atoms were placed in calculated positions and allowed to ride on their carrier atoms: C—H = 0.93–0.98 Å with *U*_iso_ = 1.5*U*_eq_(C-meth­yl) and 1.2*U*_eq_(C) for other H atoms.

## Supplementary Material

Crystal structure: contains datablock(s) global, I. DOI: 10.1107/S2056989018005455/su5434sup1.cif

Structure factors: contains datablock(s) I. DOI: 10.1107/S2056989018005455/su5434Isup2.hkl

CCDC reference: 1835595

Additional supporting information:  crystallographic information; 3D view; checkCIF report

## Figures and Tables

**Table 1:** Hydrogen-bond geometry (Å, °)

*D*—H⋯*A*	*D*—H	H⋯*A*	*D*⋯*A*	*D*—H⋯*A*
C22—H22⋯O2	0.93	2.57	3.227 (3)	128
C38—H38*A*⋯O1	0.97	2.46	3.135 (3)	127
C37—H37*A*⋯O2^i^	0.97	2.38	3.159 (3)	137
N2—H2⋯O2^i^	0.88 (3)	2.50 (2)	2.911 (3)	109.0 (19)

Symmetry code: (i) 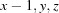.

**Table 2:** Experimental details

Crystal data	
Chemical formula	C_32_H_26_ClN_3_O_2_
*M*_r_	520.01
Crystal system, space group	Triclinic, *P* 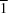
Temperature (K)	293
*a*, *b*, *c* (Å)	6.7722 (4), 11.5017 (8), 16.6305 (11)
α, β, γ (°)	80.224 (3), 84.618 (3), 81.077 (3)
*V* (Å^3^)	1258.09 (14)
*Z*	2
Radiation type	Mo *K*α
μ (mm^−1^)	0.19
Crystal size (mm)	0.23 × 0.21 × 0.19

Data collection	
Diffractometer	Bruker Kappa APEXII
Absorption correction	Multi-scan (*SADABS*; Bruker, 2004 ▸)
*T*_min_, *T*_max_	0.967, 0.974
No. of measured, independent and observed [*I* > 2σ(*I*)] reflections	25368, 4659, 3577
*R*_int_	0.035
(sin θ/λ)_max_ (Å^−1^)	0.606

Refinement	
*R*[*F* ^2^ > 2σ(*F* ^2^)], *wR*(*F* ^2^), *S*	0.047, 0.133, 1.05
No. of reflections	4659
No. of parameters	347
H-atom treatment	H atoms treated by a mixture of independent and constrained refinement
Δρ_max_, Δρ_min_ (e Å^−3^)	0.37, −0.46

Computer programs: *APEX2* and *SAINT* (Bruker, 2004 ▸), *SHELXS97* (Sheldrick, 2008 ▸), *SHELXL97* (Sheldrick, 2008 ▸) and *PLATON* (Spek, 2009 ▸).

## supplementary crystallographic information

### Crystal data

**Table:**

C_32_H_26_ClN_3_O_2_	*Z* = 2
*M**_r_* = 520.01	*F*(000) = 544
Triclinic, *P*1	*D*_x_ = 1.373 Mg m^−^^3^
Hall symbol: -P 1	Mo *K*α radiation, λ = 0.71073 Å
*a* = 6.7722 (4) Å	Cell parameters from 4659 reflections
*b* = 11.5017 (8) Å	θ = 2–26°
*c* = 16.6305 (11) Å	µ = 0.19 mm^−^^1^
α = 80.224 (3)°	*T* = 293 K
β = 84.618 (3)°	Block, colourless
γ = 81.077 (3)°	0.23 × 0.21 × 0.19 mm
*V* = 1258.09 (14) Å^3^	

### Data collection

**Table:**

Bruker Kappa APEXII diffractometer	4659 independent reflections
Radiation source: fine-focus sealed tube	3577 reflections with *I* > 2σ(*I*)
Graphite monochromator	*R*_int_ = 0.035
Detector resolution: 0 pixels mm^-1^	θ_max_ = 25.5°, θ_min_ = 2.0°
ω and φ scans	*h* = −8→8
Absorption correction: multi-scan (SADABS; Bruker, 2004)	*k* = −13→13
*T*_min_ = 0.967, *T*_max_ = 0.974	*l* = −20→20
25368 measured reflections	

### Refinement

**Table:**

Refinement on *F*^2^	Primary atom site location: structure-invariant direct methods
Least-squares matrix: full	Secondary atom site location: difference Fourier map
*R*[*F*^2^ > 2σ(*F*^2^)] = 0.047	Hydrogen site location: inferred from neighbouring sites
*wR*(*F*^2^) = 0.133	H atoms treated by a mixture of independent and constrained refinement
*S* = 1.05	*w* = 1/[σ^2^(*F*_o_^2^) + (0.057*P*)^2^ + 0.8008*P*] where *P* = (*F*_o_^2^ + 2*F*_c_^2^)/3
4659 reflections	(Δ/σ)_max_ < 0.001
347 parameters	Δρ_max_ = 0.37 e Å^−^^3^
0 restraints	Δρ_min_ = −0.46 e Å^−^^3^

### Special details

**Table:**

Geometry. All esds (except the esd in the dihedral angle between two l.s. planes) are estimated using the full covariance matrix. The cell esds are taken into account individually in the estimation of esds in distances, angles and torsion angles; correlations between esds in cell parameters are only used when they are defined by crystal symmetry. An approximate (isotropic) treatment of cell esds is used for estimating esds involving l.s. planes.
Refinement. Refinement of F^2^ against ALL reflections. The weighted R-factor wR and goodness of fit S are based on F^2^, conventional R-factors R are based on F, with F set to zero for negative F^2^. The threshold expression of F^2^ > 2sigma(F^2^) is used only for calculating R-factors(gt) etc. and is not relevant to the choice of reflections for refinement. R-factors based on F^2^ are statistically about twice as large as those based on F, and R- factors based on ALL data will be even larger.

### Fractional atomic coordinates and isotropic or equivalent isotropic displacement parameters (Å^2^)

**Table:**

	*x*	*y*	*z*	*U*_iso_*/*U*_eq_	
C2	0.2424 (3)	0.02521 (19)	0.28318 (12)	0.0341 (5)	
C3	0.2874 (3)	0.15629 (19)	0.28084 (12)	0.0308 (4)	
C4	0.4045 (3)	0.1455 (2)	0.36013 (12)	0.0346 (5)	
H4	0.5413	0.1597	0.3411	0.041*	
C5	0.4193 (4)	0.0154 (2)	0.39905 (14)	0.0439 (6)	
H5A	0.5457	−0.0115	0.4239	0.053*	
H5B	0.3102	0.0026	0.4402	0.053*	
C21	0.2282 (3)	−0.01793 (19)	0.20329 (13)	0.0354 (5)	
C22	0.3670 (4)	−0.0337 (2)	0.13896 (15)	0.0456 (6)	
H22	0.4960	−0.0160	0.1401	0.055*	
C23	0.3124 (4)	−0.0761 (2)	0.07238 (16)	0.0545 (7)	
H23	0.4052	−0.0861	0.0283	0.065*	
C24	0.1226 (4)	−0.1037 (2)	0.07072 (16)	0.0544 (7)	
H24	0.0882	−0.1317	0.0254	0.065*	
C25	−0.0169 (4)	−0.0903 (2)	0.13520 (16)	0.0487 (6)	
H25	−0.1452	−0.1093	0.1343	0.058*	
C26	0.0384 (3)	−0.04795 (19)	0.20120 (13)	0.0364 (5)	
C27	0.0290 (3)	0.01083 (19)	0.32584 (13)	0.0366 (5)	
C31	0.4322 (3)	0.18485 (19)	0.20612 (12)	0.0321 (5)	
C6	0.3840 (4)	−0.1718 (2)	0.35516 (18)	0.0606 (7)	
H03A	0.3757	−0.2075	0.3077	0.091*	
H03B	0.2642	−0.1783	0.3904	0.091*	
H03C	0.4979	−0.2120	0.3840	0.091*	
C32	0.3517 (3)	0.25452 (19)	0.13036 (12)	0.0317 (4)	
C33	0.4809 (3)	0.2861 (2)	0.06304 (13)	0.0402 (5)	
H33	0.6185	0.2756	0.0679	0.048*	
C34	0.4035 (3)	0.3327 (2)	−0.01031 (13)	0.0438 (6)	
H34	0.4870	0.3581	−0.0554	0.053*	
C35	0.1978 (3)	0.34183 (19)	−0.01695 (12)	0.0340 (5)	
C36	0.1461 (3)	0.28141 (17)	0.12155 (12)	0.0292 (4)	
C37	0.0024 (3)	0.26574 (19)	0.19474 (12)	0.0322 (5)	
H37A	−0.0604	0.1955	0.1945	0.039*	
H37B	−0.1020	0.3341	0.1916	0.039*	
C38	0.1032 (3)	0.25252 (19)	0.27456 (12)	0.0316 (4)	
H38A	0.0069	0.2330	0.3199	0.038*	
H38B	0.1434	0.3283	0.2794	0.038*	
C41	0.3264 (3)	0.2350 (2)	0.41643 (12)	0.0355 (5)	
C42	0.3936 (3)	0.3452 (2)	0.40547 (14)	0.0418 (5)	
C43	0.3308 (4)	0.4279 (2)	0.45654 (16)	0.0517 (6)	
H43	0.3777	0.5012	0.4465	0.062*	
C44	0.1979 (4)	0.4016 (3)	0.52262 (16)	0.0548 (7)	
H44	0.1572	0.4560	0.5585	0.066*	
C45	0.1265 (4)	0.2950 (3)	0.53485 (15)	0.0556 (7)	
H45	0.0353	0.2773	0.5790	0.067*	
C46	0.1880 (3)	0.2129 (2)	0.48247 (14)	0.0444 (6)	
H46	0.1356	0.1413	0.4916	0.053*	
C51	0.1096 (3)	0.37258 (19)	−0.09725 (13)	0.0364 (5)	
C52	0.2260 (4)	0.3562 (2)	−0.16886 (14)	0.0507 (6)	
H52	0.3628	0.3303	−0.1668	0.061*	
C53	0.1402 (5)	0.3779 (3)	−0.24306 (15)	0.0594 (7)	
H53	0.2192	0.3664	−0.2907	0.071*	
C54	−0.0620 (5)	0.4166 (3)	−0.24678 (15)	0.0588 (7)	
H54	−0.1200	0.4305	−0.2967	0.071*	
C55	−0.1772 (4)	0.4346 (2)	−0.17684 (15)	0.0515 (6)	
H55	−0.3135	0.4619	−0.1795	0.062*	
C56	−0.0939 (3)	0.4129 (2)	−0.10239 (14)	0.0413 (5)	
H56	−0.1743	0.4253	−0.0553	0.050*	
N1	0.4056 (3)	−0.04573 (17)	0.32992 (11)	0.0408 (4)	
N2	−0.0728 (3)	−0.03406 (17)	0.27448 (12)	0.0399 (4)	
N3	0.0704 (2)	0.32058 (15)	0.04888 (10)	0.0322 (4)	
O1	−0.0328 (2)	0.03246 (16)	0.39312 (10)	0.0503 (4)	
O2	0.6092 (2)	0.14584 (15)	0.20669 (10)	0.0455 (4)	
Cl1	0.56220 (11)	0.38517 (7)	0.32331 (5)	0.0654 (2)	
H2	−0.202 (4)	−0.037 (2)	0.2839 (16)	0.059 (8)*	

### Atomic displacement parameters (Å^2^)

**Table:**

	*U*^11^	*U*^22^	*U*^33^	*U*^12^	*U*^13^	*U*^23^
C2	0.0293 (10)	0.0390 (12)	0.0336 (11)	−0.0051 (9)	−0.0064 (8)	−0.0024 (9)
C3	0.0238 (9)	0.0399 (11)	0.0288 (10)	−0.0040 (8)	−0.0060 (8)	−0.0039 (8)
C4	0.0265 (10)	0.0461 (12)	0.0321 (11)	−0.0048 (9)	−0.0062 (8)	−0.0068 (9)
C5	0.0437 (12)	0.0480 (14)	0.0381 (12)	0.0033 (10)	−0.0142 (10)	−0.0041 (10)
C21	0.0349 (11)	0.0349 (11)	0.0365 (11)	−0.0025 (9)	−0.0064 (9)	−0.0058 (9)
C22	0.0405 (12)	0.0499 (14)	0.0487 (14)	−0.0068 (10)	−0.0001 (10)	−0.0158 (11)
C23	0.0680 (17)	0.0517 (15)	0.0463 (14)	−0.0080 (13)	0.0043 (12)	−0.0201 (12)
C24	0.0723 (18)	0.0476 (15)	0.0484 (15)	−0.0105 (13)	−0.0138 (13)	−0.0154 (12)
C25	0.0502 (14)	0.0431 (13)	0.0579 (15)	−0.0120 (11)	−0.0174 (12)	−0.0097 (11)
C26	0.0371 (11)	0.0313 (11)	0.0407 (12)	−0.0042 (9)	−0.0087 (9)	−0.0029 (9)
C27	0.0366 (11)	0.0351 (11)	0.0351 (12)	−0.0064 (9)	−0.0044 (9)	0.0050 (9)
C31	0.0233 (10)	0.0418 (12)	0.0334 (11)	−0.0073 (8)	−0.0033 (8)	−0.0094 (9)
C6	0.0669 (17)	0.0406 (14)	0.0716 (18)	0.0026 (12)	−0.0241 (14)	0.0002 (13)
C32	0.0255 (10)	0.0400 (12)	0.0311 (11)	−0.0075 (8)	0.0002 (8)	−0.0085 (9)
C33	0.0277 (10)	0.0571 (14)	0.0371 (12)	−0.0108 (10)	0.0009 (9)	−0.0083 (10)
C34	0.0382 (12)	0.0615 (15)	0.0319 (12)	−0.0167 (11)	0.0049 (9)	−0.0030 (10)
C35	0.0378 (11)	0.0358 (11)	0.0299 (11)	−0.0081 (9)	−0.0009 (9)	−0.0070 (9)
C36	0.0273 (9)	0.0314 (10)	0.0295 (10)	−0.0062 (8)	−0.0032 (8)	−0.0044 (8)
C37	0.0225 (9)	0.0412 (12)	0.0313 (11)	−0.0030 (8)	−0.0028 (8)	−0.0022 (9)
C38	0.0259 (10)	0.0391 (11)	0.0289 (10)	−0.0036 (8)	−0.0008 (8)	−0.0042 (8)
C41	0.0297 (10)	0.0457 (13)	0.0319 (11)	−0.0033 (9)	−0.0105 (8)	−0.0056 (9)
C42	0.0379 (12)	0.0531 (14)	0.0371 (12)	−0.0082 (10)	−0.0098 (9)	−0.0089 (10)
C43	0.0530 (14)	0.0519 (15)	0.0537 (15)	−0.0054 (12)	−0.0185 (12)	−0.0122 (12)
C44	0.0598 (16)	0.0609 (17)	0.0444 (14)	0.0075 (13)	−0.0139 (12)	−0.0198 (12)
C45	0.0507 (15)	0.0758 (19)	0.0382 (13)	−0.0021 (13)	0.0010 (11)	−0.0114 (12)
C46	0.0414 (12)	0.0531 (14)	0.0386 (12)	−0.0078 (11)	−0.0024 (10)	−0.0060 (11)
C51	0.0463 (12)	0.0335 (11)	0.0308 (11)	−0.0118 (9)	−0.0039 (9)	−0.0030 (9)
C52	0.0576 (15)	0.0596 (16)	0.0351 (13)	−0.0106 (12)	−0.0014 (11)	−0.0071 (11)
C53	0.080 (2)	0.0679 (18)	0.0300 (13)	−0.0144 (15)	−0.0011 (12)	−0.0050 (12)
C54	0.080 (2)	0.0622 (17)	0.0358 (14)	−0.0165 (15)	−0.0193 (13)	0.0025 (12)
C55	0.0562 (15)	0.0499 (15)	0.0478 (15)	−0.0106 (12)	−0.0181 (12)	0.0047 (11)
C56	0.0492 (13)	0.0392 (12)	0.0362 (12)	−0.0093 (10)	−0.0062 (10)	−0.0035 (9)
N1	0.0401 (10)	0.0395 (10)	0.0421 (11)	0.0021 (8)	−0.0145 (8)	−0.0053 (8)
N2	0.0318 (10)	0.0430 (11)	0.0457 (11)	−0.0109 (8)	−0.0048 (8)	−0.0027 (8)
N3	0.0324 (9)	0.0357 (9)	0.0295 (9)	−0.0060 (7)	−0.0039 (7)	−0.0060 (7)
O1	0.0482 (9)	0.0646 (11)	0.0376 (9)	−0.0161 (8)	0.0046 (7)	−0.0035 (8)
O2	0.0219 (7)	0.0657 (11)	0.0470 (9)	−0.0041 (7)	−0.0028 (6)	−0.0048 (8)
Cl1	0.0626 (4)	0.0716 (5)	0.0654 (5)	−0.0297 (4)	0.0096 (3)	−0.0096 (3)

### Geometric parameters (Å, º)

**Table:**

C2—N1	1.469 (3)	C33—H33	0.9300
C2—C21	1.511 (3)	C34—C35	1.394 (3)
C2—C27	1.569 (3)	C34—H34	0.9300
C2—C3	1.577 (3)	C35—N3	1.342 (3)
C3—C31	1.532 (3)	C35—C51	1.479 (3)
C3—C38	1.532 (3)	C36—N3	1.337 (3)
C3—C4	1.579 (3)	C36—C37	1.490 (3)
C4—C41	1.510 (3)	C37—C38	1.523 (3)
C4—C5	1.518 (3)	C37—H37A	0.9700
C4—H4	0.9800	C37—H37B	0.9700
C5—N1	1.463 (3)	C38—H38A	0.9700
C5—H5A	0.9700	C38—H38B	0.9700
C5—H5B	0.9700	C41—C42	1.389 (3)
C21—C22	1.373 (3)	C41—C46	1.391 (3)
C21—C26	1.387 (3)	C42—C43	1.375 (4)
C22—C23	1.385 (3)	C42—Cl1	1.743 (2)
C22—H22	0.9300	C43—C44	1.377 (4)
C23—C24	1.375 (4)	C43—H43	0.9300
C23—H23	0.9300	C44—C45	1.364 (4)
C24—C25	1.374 (4)	C44—H44	0.9300
C24—H24	0.9300	C45—C46	1.384 (4)
C25—C26	1.378 (3)	C45—H45	0.9300
C25—H25	0.9300	C46—H46	0.9300
C26—N2	1.391 (3)	C51—C52	1.389 (3)
C27—O1	1.210 (3)	C51—C56	1.390 (3)
C27—N2	1.358 (3)	C52—C53	1.381 (3)
C31—O2	1.213 (2)	C52—H52	0.9300
C31—C32	1.475 (3)	C53—C54	1.376 (4)
C6—N1	1.465 (3)	C53—H53	0.9300
C6—H03A	0.9600	C54—C55	1.366 (4)
C6—H03B	0.9600	C54—H54	0.9300
C6—H03C	0.9600	C55—C56	1.377 (3)
C32—C33	1.389 (3)	C55—H55	0.9300
C32—C36	1.394 (3)	C56—H56	0.9300
C33—C34	1.364 (3)	N2—H2	0.88 (3)
			
N1—C2—C21	112.06 (17)	C35—C34—H34	120.4
N1—C2—C27	113.20 (17)	N3—C35—C34	121.76 (19)
C21—C2—C27	101.09 (16)	N3—C35—C51	116.63 (18)
N1—C2—C3	102.01 (16)	C34—C35—C51	121.59 (19)
C21—C2—C3	118.75 (17)	N3—C36—C32	122.40 (18)
C27—C2—C3	110.19 (16)	N3—C36—C37	117.68 (17)
C31—C3—C38	108.09 (16)	C32—C36—C37	119.91 (17)
C31—C3—C2	107.53 (16)	C36—C37—C38	112.47 (16)
C38—C3—C2	115.01 (16)	C36—C37—H37A	109.1
C31—C3—C4	108.09 (15)	C38—C37—H37A	109.1
C38—C3—C4	114.82 (16)	C36—C37—H37B	109.1
C2—C3—C4	102.89 (15)	C38—C37—H37B	109.1
C41—C4—C5	115.76 (18)	H37A—C37—H37B	107.8
C41—C4—C3	115.84 (16)	C37—C38—C3	113.25 (17)
C5—C4—C3	105.59 (17)	C37—C38—H38A	108.9
C41—C4—H4	106.3	C3—C38—H38A	108.9
C5—C4—H4	106.3	C37—C38—H38B	108.9
C3—C4—H4	106.3	C3—C38—H38B	108.9
N1—C5—C4	103.22 (17)	H38A—C38—H38B	107.7
N1—C5—H5A	111.1	C42—C41—C46	115.9 (2)
C4—C5—H5A	111.1	C42—C41—C4	120.99 (19)
N1—C5—H5B	111.1	C46—C41—C4	123.1 (2)
C4—C5—H5B	111.1	C43—C42—C41	122.9 (2)
H5A—C5—H5B	109.1	C43—C42—Cl1	116.8 (2)
C22—C21—C26	119.4 (2)	C41—C42—Cl1	120.30 (18)
C22—C21—C2	131.5 (2)	C42—C43—C44	119.6 (3)
C26—C21—C2	109.14 (18)	C42—C43—H43	120.2
C21—C22—C23	119.2 (2)	C44—C43—H43	120.2
C21—C22—H22	120.4	C45—C44—C43	119.2 (2)
C23—C22—H22	120.4	C45—C44—H44	120.4
C24—C23—C22	120.7 (2)	C43—C44—H44	120.4
C24—C23—H23	119.6	C44—C45—C46	120.9 (2)
C22—C23—H23	119.6	C44—C45—H45	119.6
C25—C24—C23	120.8 (2)	C46—C45—H45	119.6
C25—C24—H24	119.6	C45—C46—C41	121.4 (2)
C23—C24—H24	119.6	C45—C46—H46	119.3
C24—C25—C26	118.2 (2)	C41—C46—H46	119.3
C24—C25—H25	120.9	C52—C51—C56	118.4 (2)
C26—C25—H25	120.9	C52—C51—C35	120.8 (2)
C25—C26—C21	121.8 (2)	C56—C51—C35	120.7 (2)
C25—C26—N2	128.1 (2)	C53—C52—C51	120.6 (3)
C21—C26—N2	110.08 (19)	C53—C52—H52	119.7
O1—C27—N2	125.8 (2)	C51—C52—H52	119.7
O1—C27—C2	126.6 (2)	C54—C53—C52	120.2 (2)
N2—C27—C2	107.54 (18)	C54—C53—H53	119.9
O2—C31—C32	119.56 (18)	C52—C53—H53	119.9
O2—C31—C3	121.31 (18)	C55—C54—C53	119.7 (2)
C32—C31—C3	119.01 (16)	C55—C54—H54	120.2
N1—C6—H03A	109.5	C53—C54—H54	120.2
N1—C6—H03B	109.5	C54—C55—C56	120.8 (2)
H03A—C6—H03B	109.5	C54—C55—H55	119.6
N1—C6—H03C	109.5	C56—C55—H55	119.6
H03A—C6—H03C	109.5	C55—C56—C51	120.4 (2)
H03B—C6—H03C	109.5	C55—C56—H56	119.8
C33—C32—C36	118.10 (19)	C51—C56—H56	119.8
C33—C32—C31	120.06 (18)	C5—N1—C6	112.86 (19)
C36—C32—C31	121.61 (18)	C5—N1—C2	105.95 (17)
C34—C33—C32	119.1 (2)	C6—N1—C2	114.76 (18)
C34—C33—H33	120.4	C27—N2—C26	112.06 (18)
C32—C33—H33	120.4	C27—N2—H2	120.9 (18)
C33—C34—C35	119.3 (2)	C26—N2—H2	125.6 (18)
C33—C34—H34	120.4	C36—N3—C35	118.38 (17)
			
N1—C2—C3—C31	87.78 (18)	C33—C34—C35—C51	−169.7 (2)
C21—C2—C3—C31	−35.9 (2)	C33—C32—C36—N3	9.5 (3)
C27—C2—C3—C31	−151.73 (16)	C31—C32—C36—N3	−164.92 (19)
N1—C2—C3—C38	−151.78 (16)	C33—C32—C36—C37	−170.98 (19)
C21—C2—C3—C38	84.5 (2)	C31—C32—C36—C37	14.6 (3)
C27—C2—C3—C38	−31.3 (2)	N3—C36—C37—C38	−165.11 (18)
N1—C2—C3—C4	−26.19 (19)	C32—C36—C37—C38	15.4 (3)
C21—C2—C3—C4	−149.88 (17)	C36—C37—C38—C3	−51.8 (2)
C27—C2—C3—C4	94.30 (18)	C31—C3—C38—C37	55.5 (2)
C31—C3—C4—C41	117.6 (2)	C2—C3—C38—C37	−64.7 (2)
C38—C3—C4—C41	−3.1 (3)	C4—C3—C38—C37	176.20 (16)
C2—C3—C4—C41	−128.80 (18)	C5—C4—C41—C42	147.3 (2)
C31—C3—C4—C5	−112.83 (19)	C3—C4—C41—C42	−88.3 (2)
C38—C3—C4—C5	126.44 (19)	C5—C4—C41—C46	−31.5 (3)
C2—C3—C4—C5	0.7 (2)	C3—C4—C41—C46	93.0 (2)
C41—C4—C5—N1	154.80 (17)	C46—C41—C42—C43	0.7 (3)
C3—C4—C5—N1	25.2 (2)	C4—C41—C42—C43	−178.2 (2)
N1—C2—C21—C22	−55.7 (3)	C46—C41—C42—Cl1	−178.29 (16)
C27—C2—C21—C22	−176.6 (2)	C4—C41—C42—Cl1	2.8 (3)
C3—C2—C21—C22	62.8 (3)	C41—C42—C43—C44	1.1 (4)
N1—C2—C21—C26	122.16 (19)	Cl1—C42—C43—C44	−179.90 (18)
C27—C2—C21—C26	1.3 (2)	C42—C43—C44—C45	−1.9 (4)
C3—C2—C21—C26	−119.2 (2)	C43—C44—C45—C46	0.9 (4)
C26—C21—C22—C23	1.7 (3)	C44—C45—C46—C41	1.0 (4)
C2—C21—C22—C23	179.5 (2)	C42—C41—C46—C45	−1.7 (3)
C21—C22—C23—C24	−0.7 (4)	C4—C41—C46—C45	177.1 (2)
C22—C23—C24—C25	−0.3 (4)	N3—C35—C51—C52	−157.6 (2)
C23—C24—C25—C26	0.4 (4)	C34—C35—C51—C52	21.0 (3)
C24—C25—C26—C21	0.7 (3)	N3—C35—C51—C56	19.1 (3)
C24—C25—C26—N2	−175.9 (2)	C34—C35—C51—C56	−162.3 (2)
C22—C21—C26—C25	−1.8 (3)	C56—C51—C52—C53	−0.9 (4)
C2—C21—C26—C25	−180.0 (2)	C35—C51—C52—C53	175.9 (2)
C22—C21—C26—N2	175.4 (2)	C51—C52—C53—C54	0.2 (4)
C2—C21—C26—N2	−2.8 (2)	C52—C53—C54—C55	0.7 (4)
N1—C2—C27—O1	58.7 (3)	C53—C54—C55—C56	−0.9 (4)
C21—C2—C27—O1	178.8 (2)	C54—C55—C56—C51	0.2 (4)
C3—C2—C27—O1	−54.8 (3)	C52—C51—C56—C55	0.7 (3)
N1—C2—C27—N2	−119.41 (19)	C35—C51—C56—C55	−176.1 (2)
C21—C2—C27—N2	0.6 (2)	C4—C5—N1—C6	−170.69 (19)
C3—C2—C27—N2	127.08 (18)	C4—C5—N1—C2	−44.3 (2)
C38—C3—C31—O2	158.3 (2)	C21—C2—N1—C5	172.38 (18)
C2—C3—C31—O2	−77.0 (2)	C27—C2—N1—C5	−74.1 (2)
C4—C3—C31—O2	33.5 (3)	C3—C2—N1—C5	44.3 (2)
C38—C3—C31—C32	−25.8 (2)	C21—C2—N1—C6	−62.4 (3)
C2—C3—C31—C32	98.9 (2)	C27—C2—N1—C6	51.1 (3)
C4—C3—C31—C32	−150.66 (18)	C3—C2—N1—C6	169.51 (19)
O2—C31—C32—C33	−6.9 (3)	O1—C27—N2—C26	179.5 (2)
C3—C31—C32—C33	177.10 (19)	C2—C27—N2—C26	−2.4 (2)
O2—C31—C32—C36	167.4 (2)	C25—C26—N2—C27	−179.7 (2)
C3—C31—C32—C36	−8.6 (3)	C21—C26—N2—C27	3.4 (3)
C36—C32—C33—C34	−5.4 (3)	C32—C36—N3—C35	−4.4 (3)
C31—C32—C33—C34	169.2 (2)	C37—C36—N3—C35	176.14 (18)
C32—C33—C34—C35	−3.3 (4)	C34—C35—N3—C36	−4.9 (3)
C33—C34—C35—N3	8.8 (4)	C51—C35—N3—C36	173.66 (18)

### Hydrogen-bond geometry (Å, º)

**Table:**

*D*—H···*A*	*D*—H	H···*A*	*D*···*A*	*D*—H···*A*
C22—H22···O2	0.93	2.57	3.227 (3)	128
C38—H38*A*···O1	0.97	2.46	3.135 (3)	127
C37—H37*A*···O2^i^	0.97	2.38	3.159 (3)	137
N2—H2···O2^i^	0.88 (3)	2.50 (2)	2.911 (3)	109.0 (19)

Symmetry code: (i) *x*−1, *y*, *z*.
